# The complement cascade in lung injury and disease

**DOI:** 10.1186/s12931-023-02657-2

**Published:** 2024-01-04

**Authors:** M. G. Detsika, K. Palamaris, I. Dimopoulou, A. Kotanidou, S. E. Orfanos

**Affiliations:** 1 1st Department of Critical Care Medicine & Pulmonary Services, GP Livanos and M Simou Laboratories, Evangelismos Hospital, National and Kapodistrian University of Athens, 3, Ploutarchou St., 10675 Athens, Greece; 2 1st Department of Pathology, School of Medicine, National and Kapodistrian University of Athens, Athens, Greece

**Keywords:** Complement, Lung disease, Complement-related therapeutics

## Abstract

**Background:**

The complement system is an important arm of immune defense bringing innate and adaptive immunity. Although originally regarded as a major complementary defense mechanism against pathogens, continuously emerging evidence has uncovered a central role of this complex system in several diseases including lung pathologies.

**Main body:**

Complement factors such as anaphylatoxins C3a and C5a, their receptors C3aR, C5aR and C5aR2 as well as complement inhibitory proteins CD55, CD46 and CD59 have been implicated in pathologies such as the acute respiratory distress syndrome, pneumonia, chronic obstructive pulmonary disease, asthma, interstitial lung diseases, and lung cancer. However, the exact mechanisms by which complement factors induce these diseases remain unclear. Several complement-targeting monoclonal antibodies are reported to treat lung diseases.

**Conclusions:**

The complement system contributes to the progression of the acute and chronic lung diseases. Better understanding of the underlying mechanisms will provide groundwork to develop new strategy to target complement factors for treatment of lung diseases.

Lung diseases remain the third most common cause of death globally [1]. They range from acute forms of lung disease such as pneumonia and lower respiratory infections to chronic respiratory diseases, such as chronic obstructive pulmonary disease (COPD), interstitial lung diseases (ILDs) and lung cancer. Understanding the underlying mechanisms driving lung diseases is therefore crucial in order to design and apply successful treatment strategies and carefully plan prevention and control. The current review article describes in detail the role of an innate immunity machinery, the complement system, in the various forms of lung disease highlighting its involvement in disease mechanisms and progression and addressing its potential as a target for future treatment strategies in order to combat the burden of lung disease.

## The complement system

**Fig. 1:**
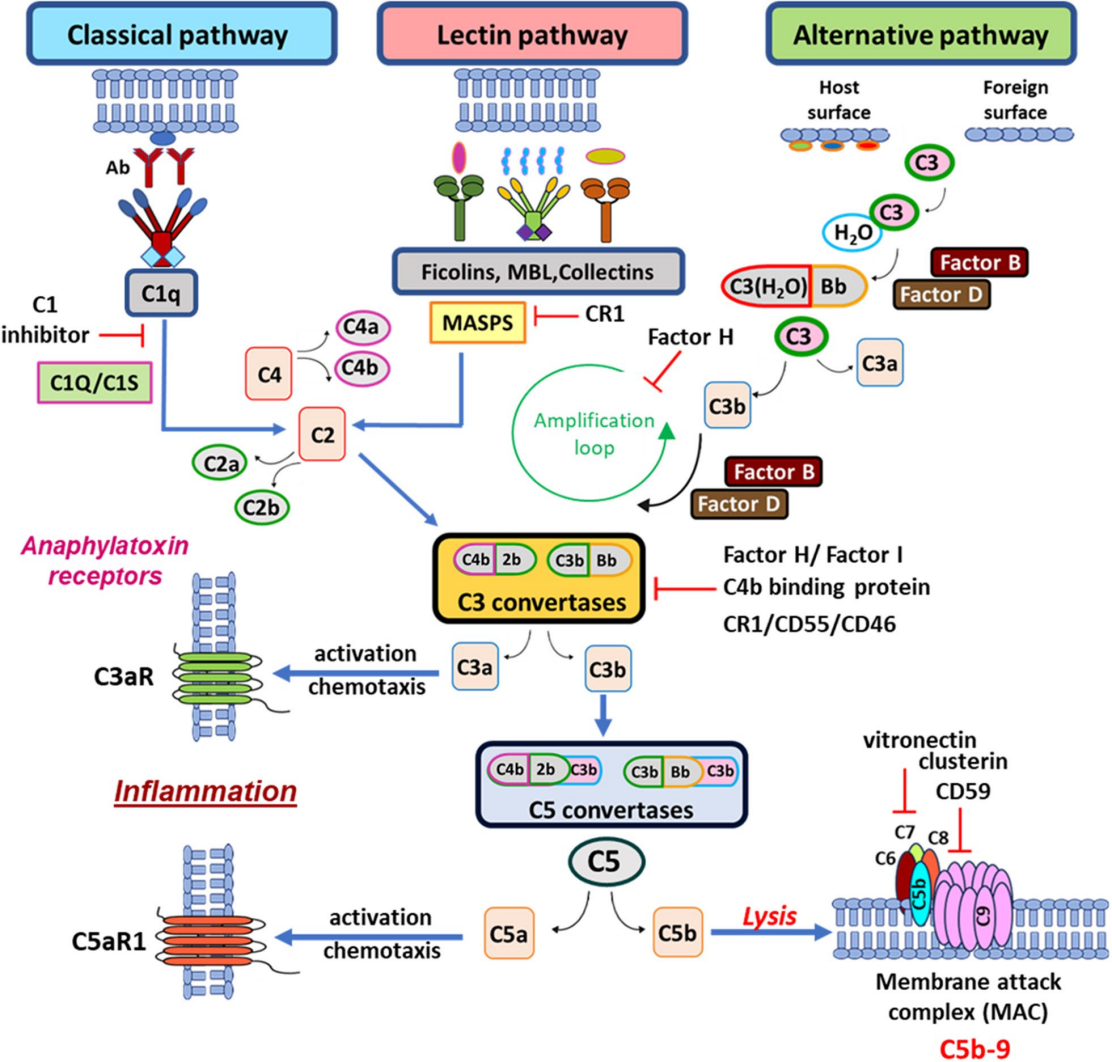
The complement cascade pathways. The classical pathway is initiated by binding of C1q to antibody chains triggering the activation of C1s and C1r. The activated C1 complex cleaves circulating C2 and C4 molecules into active C2a, C2b and C4a, C4b molecules leading to the formation of the C3 convertase following binding of the active C2b to C4b (C4b2b). The lectin pathway is triggered by recognition of microbial carbohydrates by ficolins, mannan binding lectin (MBL), or collectins which bind to mannan binding lectin serine peptidases (MASPs) forming a complex. The activated MASPs also lead to cleavage of C2 and C4 into active C2a, C2b and C4a, C4b fragments and thus the formation of the C3 convertase (C4b2b). The alternative pathway is triggered by spontaneous C3 activation by C3 hydrolysis which in the presence of Factor B and Factor D leads to the formation of a fluid phase C3 convertase through binding of the hydrolysed C3 to an active Bb fragment (C3(H2O)Bb. C3(H2O)Bb has the ability to cleave C3 into active C3a and C3b fragments. Binding of C3b to Bb results in the formation of the C3 convertase of the alternative pathway (C3bBb). All three pathways converge at the C3 step in order for the cascade to proceed further to the formation of the C5 convertase by binding of an active C3b molecule to the existing C3 convertases which results in their conversion into active C5 convertases (C4b2bC3b, C3bBbC3b). The C5 convertases cleave circulating C5 molecules into active C5a and C5b molecules allowing binding of C5b to the cell membrane and its assembly with C6, C7 and C8 molecules in order to polymerize C9 molecules  and eventually form C5b-9, also known as the membrane attack complex (MAC). Formation of sufficient MAC molecules on the cell membrane ultimately leads to lysis while simultaneous release of C3a and C5a anaphylatoxins and their binding to respective cellular receptors C3aR and C5aR1, will allow for initiation of cellular activation and chemotaxis promoting a strong inflammatory state. The presence of complement regulatory proteins (CRPs), cell bound (CD55, CD46, CD59, CR1) or circulating (C4b binding protein, Factor H, vitronectin, clusterin) prevent overactivation of the complement cascade pathways and dysregulation of the complement system

The complement is an important innate immune surveillance system constantly in search of pathogens in order to act for their effective removal and destruction, while simultaneously triggering inflammatory responses thus adding to the overall immune response and maintaining cellular homeostasis. The complement system includes more than 30 plasma and membrane-bound proteins [2, 3] which are produced in the liver or are cellular membrane proteins and are involved in the activation of the complement cascade pathways. There are three pathways of complement activation, the classical, the alternative and the lectin pathway all leading to the activation of the terminal pathway depicted in Fig. 1.

### Classical pathway

Classical pathway activation is initiated by binding of immunoglobulins IgM or IgG to their target antigen either on the pathogen cell membrane or on immune complexes resulting to the exposure of a C1q binding site. Binding of the C1q subunit of the C1 complex, which also contains two C1r and C1s serine protease subunits, allows the activation of C1r and C1s. The activated C1s subsequently cleaves C2 and C4 into C2a, C2b and C4a and C4b active fragments, respectively. Binding of a C4b fragment to C2b leads to the formation of the classical pathway C3 convertase (C4b2b) (Fig. 1) [3–5].

### Lectin pathway

Activation of the lectin pathway is mediated by pattern recognition proteins (PRPs) mannan-binding lectin (MBL), ficolins (ficolin-1, ficolin-2, and ficolin-3) and collectins (collectin-1) which have the ability to bind to various carbohydrate ligands present on the surface of microorganisms. Each of the above-mentioned molecules combine with MBL-associated serine proteases (MASPs) (MASP-1, MASP-2 and MASP-3) forming a complex similar to the C1 complex of the classical complement pathway, in which MASPs resemble the C1s and C1r units. MASP1 activates MASP2 which has the ability to cleave C4 and subsequently C2, allowing for binding of C4b to C2b and the formation of the lectin pathway C3 convertase (C4b2b) (Fig. 1) [3–5].

### Alternative pathway

Activation of the alternative pathway is subject to spontaneous hydrolysis of C3 in plasma, leading to the formation of C3(H2O). In the presence of Factor B and Factor D the hydrolysed C3 binds to an active Bb fragment thus generating C3(H2O)Bb, a fluid phase C3 convertase which cleaves C3 into active C3a and C3b. The active C3b molecule then binds on the cell membrane and subsequent binding to Bb enables the generation of the active C3 convertase C3bBb which also cleaves C3 into new C3a and C3b molecules. The fluid phase C3 convertase containing the hydrolysed C3 has a lower activity than C3bBb, which is twice more active, but is more stable and resistant to inactivation (Fig. 1) [3–6].

The three pathways converge at the C3 step at which the C3 convertase is assembled and constantly cleaves additional C3 to C3a and C3b active fragments. The same C3 convertase, namely C4b2b, is generated in the classical and lectin pathways, whereas in the alternative pathway a different C3 convertase (C3bBb) is generated. The addition of an active C3b molecule to any of the C3 convertases results in loss of their ability to cleave C3 and switches them into a C5 convertase. The latter cleaves C5 into C5a and C5b active fragments. This step allows for release of C5a and the subsequent assembly of the membrane attack complex (MAC) by binding of C5b to C6, C7, and C8 thus permitting the polymerization of C9 fragments in order to form C5b-9 (MAC). Formation of the MAC can lead to cell lysis (Fig. 1) [3–5]. C3a and C5a molecules are also known as anaphylatoxins [7]. Upon binding to their receptors, C3aR, C5aR1 and C5aR2, they may induce a series of inflammatory events such as recruitment of neutrophils, eosinophils, monocytes, and T lymphocytes, activation of phagocytic cells with concomitant release of granule-based enzymes and generation of oxidants, all of which contribute to innate immune functions or tissue damage [7, 8].

As the complement system may also attack host cells, complement regulatory mechanisms have evolved in order to restrict complement activity to designated targets. The complement system is comprised by highly labile components that are inactivated spontaneously if they are not stabilized through a reaction with other components. In addition, a series of proteins which regulate the activation of the complement cascade can inactivate various complement components thus ensuring the balanced activation of all pathways. However, excessive complement activation can still occur if complement activation has exceeded the capacity of host control [2]. Uncontrolled complement activation can occur in the host under pathological conditions and may be harmful due to induction and augmentation of inflammation [5, 9, 10].

### Complement regulatory proteins (CRegPs)

**Fig. 2:**
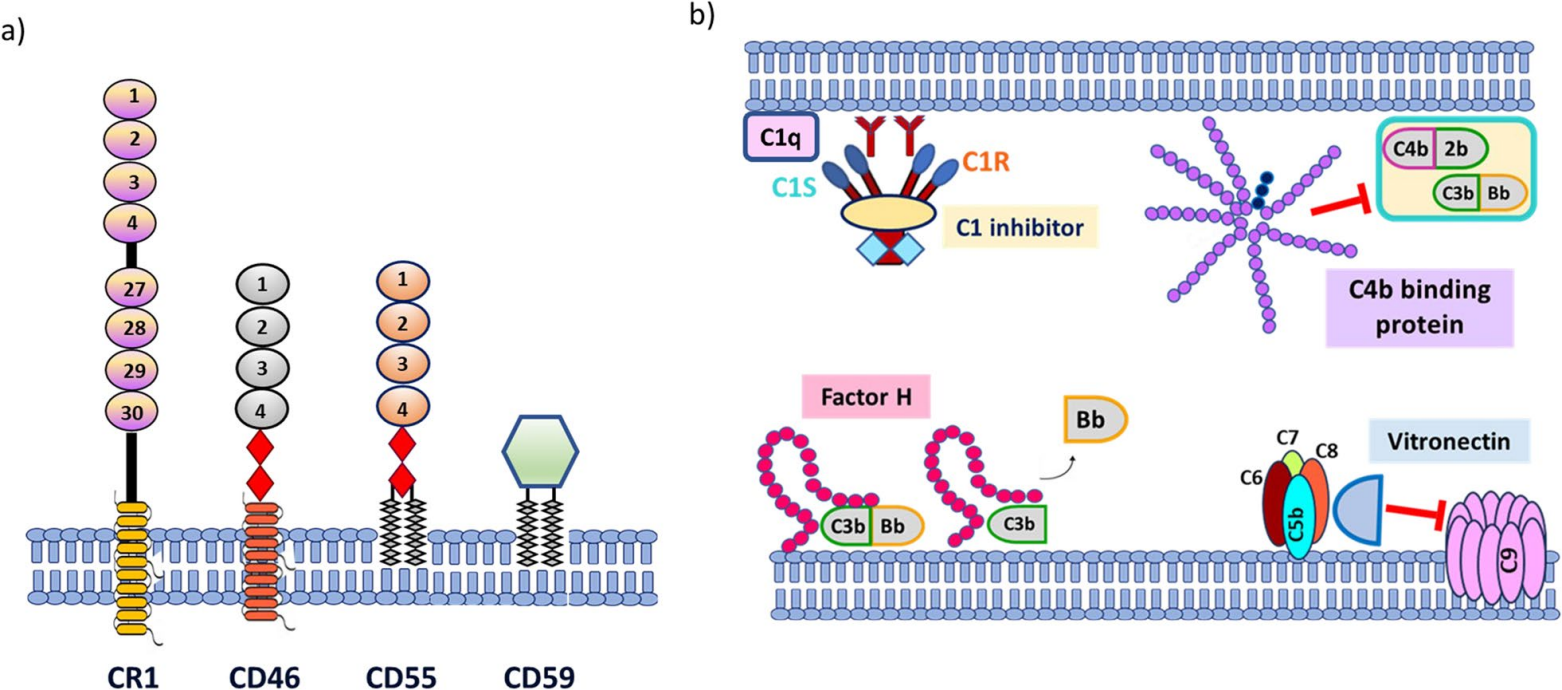
Complement regulatory proteins (CRegPs) in humans. **a** Membrane bound CRegPs share similar structural characteristics facilitating their correct function. CR1 and CD46 attach on the membrane via a transmembrane domain while CD55 and CD59 are attached via a glycosylphosphatidylinositol-(gpi) anchor. Their extracellular domains consist of multiple short consensus repeats (SCR) as shown for CR1, CD46 and CD55 while CD59 has a simpler extracellular domain consisting only of aminoacids linked by disulphide bonds. CD46 and CD55 also share a common serine-threonine-rich structure between their attachment domain and the short consensus repeats. **b** Structural characteristics of fluid phase complement regulatory proteins. C1 inhibitor, a serine protease inhibitor (serpin) consists of two domains, a C-terminal serpin domain and a N-terminal non-serpin domain the latter important for functionality and recognition. C4b binding protein, is a spider-like structure which consists of seven identical α-chains and one β-chain. The α-chain is comprised of eight SCRs and a C-terminal oligomerization domain, while the β-chain consists of three short consensus repeats and a C-terminal oligomerization domain. Factor H is a glycoprotein consisting of 20 short consensus repeats and its activity is regulated by the first four short consensus repeats while consensus repeats 19–20 are involved in ligand binding. Vitronectin is a glycoprotein with a complex structure enabling its multifunctional character and in humans it circulates either as a single chain of 75 kDa, or a clipped form of two chains: 65 and 10 kDa, held together by a disulphide bond

In order to counteract the possible cellular damage caused by uncontrolled complement activity, various mechanisms exist that regulate complement activation and maintain a well-balanced response of the complement cascade to various triggers. They involve the expression of specific protein molecules with the potential to restrict uncontrolled activation by eliminating the activity of effector proteins depicted in Fig. 2 [11]. CRegPs are found both in the fluid phase [12, 13] as well as on the cell surface [14] (Fig. 2). In humans, membrane bound complement control proteins include complement receptor type 1 (CR1), CD46 or membrane co-factor protein (MCP), CD55 and CD59 protein molecules (Fig. 2a). Their function involves acceleration of the dissociation of C3 and/or C5 convertases such as in the case of CD55 [15, 16], or prevention of C5a binding and assembly of the MAC such as in the case of CD59 which acts exclusively in the final step of the cascade thus preventing cell lysis [17, 18] (Fig. 1). MCP acts as a co-factor for factor I-mediated degradation of C3b and C4b [14, 19] (Fig. 1). CR1 competes with MASPs for binding of MBL and ficolins thus inhibiting the initiation of the lectin pathway (Fig. 1) [14]. Furthermore, upon binding, CR1 either internalizes the opsonized elements or presents them to other immune cells, thus blocking C5 convertase formation (Fig. 1) [20]. CR1 may also act as a cofactor for the Factor I-mediated cleavage of soluble/bound C3b and C4b, thus eliminating extreme complement activation (Fig. 1) [20, 21]. Fluid phase plasma complement regulatory proteins are: C4b binding protein [22–24], C1 inhibitor [25, 26], Factor H [27–29] and vitronectin [30, 31] (Fig. 2b). C4b binding protein binds to C3 convertases and facilitates their dissociation [32], while Factor H binds to the alternative pathway C3 convertase and allows its dissociation with subsequent release of Bb (Fig. 1) [33]. C1 inhibitor binds to C1q and prevents C1s and C1r activation thus preventing C3 convertase activation [25] and vitronectin is known to prevent the final step of the complement pathway by prevention of MAC assembly (Fig. 1) [31].

### Complement activation in the lung

As the lung is a target for microbial and viral infections, the activation of the complement system would be regarded as a promising defense mechanism. Indeed, previous evidence has shown that activation of the complement system takes place in the lung, since the presence of complement factors has been detected in the bronchoalveolar lavage fluid (BALF) of healthy individuals [34–36], while a recent single cell RNA-seq study identified the expression of numerous complement genes including *C5, C1q*, *FCN1, CFI, C6 and C7* in type II alveolar epithelial cells and interstitial lung macrophages [37]. Detection of complement factors in the lung has also been reported in pulmonary alveolar type II epithelial cells of mice [38] as well as in BALF of rats [39], baboons [40] and rabbits [41]. The finding of local complement activation emphasizes a possibly important role of the complement system in fighting off infections, but also suggests that the complement system may generally affect lung injury and be involved in lung disease mechanisms. A thorough description of the putative effect of complement system activation in major lung pathologies follows.

## Acute lung pathologies

### Acute respiratory distress syndrome (ARDS)

The acute respiratory distress syndrome is a life-threatening condition accounting for 10% of the admissions to intensive care units. It was originally described by Ashbaugh in 1967 [42] and obtained its initial definition by the American-European Consensus Conference in 1994 [43]. Since then, it has been redefined as a type of acute diffuse, inflammatory lung injury, which leads to augmented pulmonary vascular permeability, increased lung weight, and loss of aerated lung tissue [44] and it is categorized into mild, moderate and severe ARDS according to arterial oxygen pressure/fraction of inspired oxygen (PaO_2_/FIO_2_) and positive end-expiratory pressure (PEEP) measurements [44].

The exact involvement of complement in the pathobiology of ARDS remains to be determined. A recent study identified the key role of the lectin pathway in lipopolysaccharide (LPS) mediated ARDS in mice [45]. The study showed that administration of HG-4, a monoclonal antibody that targets MASP-2 and inhibits lectin pathway activation decreased levels of myeloid peroxide, lactate dehydrogenase (LDH), tumor necrosis factor-α (TNF-α), and interleukin (IL)-6, and reduced mortality of mice receiving LPS [45]. Furthermore, studies have highlighted the role of C5a anaphylatoxin in the recruitment of neutrophils in the lung resulting in tissue damage and subsequent injury. Initial studies on a primate sepsis model by *Escherichia coli* infusion, revealed that administration of a C5a inhibitor resulted in prevention of mortality, amelioration of ARDS and acceleration of recovery [46] while a subsequent study on the same animal model with a C5a inhibitor administration confirmed the reduction of mortality and pulmonary edema, as well as the reduction of C5a levels in the treated animals, indicating the important role of C5a in ARDS development and progression [47]. Efforts to delineate the effect of the C5a-C5a-receptors (C5aR1 and C5aR2)-axis effect on ARDS, have been based mainly on studies involving mouse and rat models of LPS-induced lung inflammation/injury, or immune complexes-mediated lung injury, with variable and conflicting results. Muligan et al. utilized ARDS models associated with immune complex formation and complement activation by cobra venom factor in rats and demonstrated the requirement of C5a for the generation of the full inflammatory response [48]. On the other hand, a subsequent study which used complement deficient (*C3*^*−/−*^ and *C5*^*−/−*^) mouse models of LPS-induced ARDS reported that the LPS-induced lung injury was independent from complement, since no significant differences were observed between *C3*^*−/−*^, *C5*^*−/−*^ and wild type (WT) mice [49]. However, the same group identified a crucial role of C5aR1 in the same model of LPS induced ARDS in mice [50] as well as in a model of immune complexes induced ARDS; the latter finding was initially reported earlier in a study which used a gene targeted disruption of the C5aR1 gene and immune complex mediated ARDS in mice [51]. The role of C5aR2 in ARDS mouse models was further investigated and confirmed in a subsequent study with a mouse model of C5aR2 receptor deficiency (*C5L2*^*−/−*^) which showed that administration of LPS significantly increased airway edema and hemorrhage, as well as BALF and lung tissue neutrophil counts in *C5L2*^*−/−*^ mice in comparison with wild type animals. When a blocking antibody against the C5aR2 was administered before LPS administration, the increased neutrophilic infiltration and cytokine levels were reversed [52]. One possible mechanism for the C5a-C5aR1/2-axis effect on LPS-mediated ARDS was described by Bosmann et al. who identified the appearance of histones H3 and H4 in BALF of mice with ARDS triggered upon ligation of C5a to C5aR1 and C5aR2 [50]. Increased presence of histones was also verified in BALF samples from patients with ARDS in comparison with samples from healthy individuals in which absence of histones was reported [50].

Increased complement activation and elevated C5a levels were reported in patients at risk for ARDS due to trauma or bacteremia, and the possible role of C5a as a mediator of neutrophil accumulation was suggested by authors [53]. Subsequent observations supporting a possible role of complement in ARDS, were reported in a prospective study of intensive care unit (ICU) patients who developed ARDS, revealing abnormal C3 consumption and elevated plasma C5a activity in the majority of ARDS cases [54], while another study provided evidence of C3 activation in plasma samples and C3 and C5a activation, in BALF samples of all patients with ARDS [55]. The latter study also identified a possible link between C5a and the neutrophil influx observed in ARDS patients’ lungs [55]. Subsequent studies confirmed complement activation in ARDS patients [56, 57] and demonstrated elevated levels of C5b-9 two days prior to developing ARDS, thus identifying C5b-9 as a promising marker of ARDS prognosis. Increased complement activation was further confirmed in studies involving trauma patients who later developed ARDS, also emphasizing the prognostic value of C3a in this group of patients [58, 59]. The involvement of the alternative complement pathway activation in ARDS was also highlighted in a recent study which reported complement factor H deficiency as a key feature of patients developing ARDS [60]. The study combined measurements and data from three major clinical trials, the ARDSnet, Lisofylline and Respiratory Management of Acute Lung Injury (LARMA) trial, the Statins for Acutely Injured Lungs from Sepsis (SAILS), and the Acute Lung Injury Registry and Biospecimen Repository (ALIR) trial. Bain et al. identified a subgroup of ARDS patients with increased mortality characterized by complement exhaustion and diminished alternative pathway activation. These patients also exhibited factor H deficiency resulting to higher Ba:B and C3a:C3 ratios and lower factor B and C3 levels [60].

### Pneumonia

Pneumonia may be caused by either bacterial or viral agents. Both agents are recognized by the complement system resulting in the activation of the cascade. The role of complement has been studied using experimental disease models as well as in clinical settings and will be discussed in detail.

### Bacterial pneumonia

*Streptococcus pneumoniae* (*S. pneumoniae)*, represents the most frequent cause of bacterial pneumonia, especially in individuals with complement dysfunction due to genetic abnormalities, such as complement factor C2 or D deficiency [61, 62]. In-vitro activation of both the classical and the alternative pathways, has been reported by the pore-forming pneumococcal toxin, pneumolysin [63], and the teichoic acid of the bacterial wall respectively [64]. Activation of all three complement pathways has also been observed in-vitro by the LPS and outer membrane proteins of a hospital acquired pathogen *Klebsiella pneumoniae* (*K. pneumoniae)* [65]. The protective effect of C3 against bacterial lung infection has been reported for both *S. pneumoniae* [65] in-vitro, as well as in-vitro and in-vivo for *K. pneumoniae* [66], and in an in-vivo mouse model of *Pseudomonas aeruginosa* (*P. aeruginosa)* infection [67], all the above supporting the successful pathogen killing mechanisms of complement. These studies highlighted the importance of C3-mediated opsonization and phagocytosis in clearance of these pathogens.

Although complement activation may result in the killing of pathogens, evidence suggests that bacterial agents such as the aforementioned have evolved various properties for evading complement. Mouse models of lung infections with *S. pneumoniae* have shown the role of choline binding proteins, such as PspA and PspC, which block the classical and alternative complement pathways respectively. PspA suppresses binding of C-reactive protein (CRP) to *S. pneumoniae*, while PspC exerts its anti-complement function by binding to factor H and magnifying its potential to suppress factors B and C3b interactions. Three additional proteins, termed as histidine triad proteins (PhtA, PhtB, PhtD) are capable of inhibiting complement activation, mainly by enhancing factor H inhibitory functions [68]. Similar evasion mechanisms have been described for *K. pneumoniae.* One mechanism involves protection by the bacterial capsule, which has been shown to provide a barrier against destruction by MAC [69–71] and to eliminate C3b deposition on various clinical isolates [72, 73]. Other mechanisms involve changes in LPS composition [74, 75] and modification of outer membrane proteins [76].

Reduced levels of alternative pathway activation have been reported in patients with pneumococcal pneumonia and with normal levels of the classical and lectin pathways [77], while a subsequent study revealed increased levels of complement activation in patients with complicated disease in comparison to patients with a milder form of pneumococcal disease [78]. A recent study examined the activation of complement in patients with community acquired pneumonia (CAP) and revealed increased levels of C3a in CAP patients in comparison to healthy controls [79]. The study identified a positive correlation of C3a levels and disease severity thus highlighting the potential of C3a as an early prognostic marker for CAP, and further demonstrated that inhibition of complement, ameliorated *S. pneumoniae*-mediated secretion of inflammatory cytokines in pulmonary epithelial cells [79].

### Viral pneumonia

Complement involvement has also been observed in viral pneumonia. In a mouse model of middle east respiratory syndrome coronavirus (MERS-CοV) infection, complement overactivation and excessive cytokine production was reported with elevated levels of C5a in lung and serum samples, while inhibition of C5aR was shown to reduce viral replication and alveolar damage possibly by decreasing alveolar macrophage infiltration and interferon (IFN)-γ receptor expression [80]. Regarding severe acute respiratory syndrome coronavirus (SARS-CoV) infection, MBL has been shown to effectively detect and neutralise SARS-CoV pseudovirus by binding to SARS-CoV spike glycoprotein [81]. A previous study reported that activation of complement component C3 exacerbates disease in ARDS due to SARS-CoV infection, suggesting that C3 inhibition may also alleviate the inflammatory lung complications of such infection [82]. Activation of the complement system has also been reported by respiratory syncytial virus (RSV) infected cells in-vitro [83]*,* despite the normal levels of circulating complement factors in RSV infected individuals [84] in addition, increased levels of C3a and C5a have been reported in BALF [85] and serum samples of patients with H1N1 viral infection [86]. The levels of the same anaphylatoxins were also found elevated in BALF samples of MERS-CοV patients [87] and correlated with levels of IL-8 and regulated on activation, normal T cell expressed and secreted (RANTES) cytokine, as well as with mortality, suggesting that these molecules may contribute to disease pathogenesis and may also serve as disease prognosis markers.

In the recently emerged infection by SARS-CoV-2, Coronavirus disease (COVID-19) which quickly evolved into a pandemic, extensive investigation of the role of complement was performed both as a disease feature, as well as for its involvement in disease mechanisms. Initial studies reported that mortality of COVID-19 patients, due to COVID-19–associated respiratory failure, was associated with distinctive vascular features of the lung, such as severe endothelial injury associated with the presence of intracellular virus and disrupted cell membranes, as well as widespread thrombosis with microangiopathy in pulmonary vessels [88]. Complement activation was further reported in lung and skin biopsies of COVID-19 patients with severe disease [89], with colocalization of C5b-9 and C4d with SARS-CοV-2 spike protein in interalveolar septa and the cutaneous microvasculature. The aforementioned study identified a possible link between complement overactivation and the microvascular injury and procoagulant state observed in severe and critical cases of COVID-19, both of which have been described as specific features of COVID-19 associated viral infection [88], [89]. The pulmonary microthrombi identified in these patients were also reported to contain trapped partly degenerated neutrophils, consistent with neutrophil extracellular traps (NET)osis [90]. A possible link between complement and the platelet/NET/thrombin axis was identified by a subsequent study [91]. Using both patients’ samples and in-vitro cellular assays, Skendros et al. identified high tissue factor expression in neutrophils from COVID-19 patients which was disrupted using a C3 inhibitor [91]. Furthermore, C5aR1 blocking or netosis and thrombin inhibition in neutrophils from COVID-19 subjects was found to diminish platelet-mediated NET-driven thrombogenicity, supporting the pivotal role of complement in net-driven immunothrombosis present in COVID-19 patients [91]. Numerous studies revealed increased complement factor and fragment levels in severely and critically ill COVID-19 subjects indicative of complement overactivation [92–96] possibly contributing to the highly inflammatory state exhibited in such patients. C3a, C5a, and C5b-9 as well as C4d were found significantly increased with disease severity and correlated with specific disease characteristics and antiviral antibodies, thus rendering them into possible disease progression markers [94–97]. Carvelli et al. demonstrated an association of increasing soluble C5a levels with COVID-19 severity, as well as increased expression of C5aR in blood and myeloid cells [98]. Furthermore, using a C5aR knock-in mouse model, the study showed that administration of anti-C5aR monoclonal antibodies eliminated C5aR-mediated activation and infiltration of myeloid cells thus preventing the excessive lung inflammation and endothelialitis associated with ARDS in COVID-19 patients [98]. The role of C3 was also highlighted in another single cell RNA sequencing study, which reported that the C3 rich environment in severely ill patients promotes activation of highly cytotoxic CD16^+^ T cells, associated with poor prognosis and a fatal outcome [60]. Furthermore, in a single cell RNA sequencing study of peripheral blood mononuclear cells (PBMCs) isolated from COVID-19 patients, an increase in the expression of complement regulatory protein CD55 in monocyte, as well as, in T and B cell populations of severely and critically ill patients was detected, indicative of complement overactivation [99]. The CD55 increase observed in these distinct cell populations of COVID-19 patients was reported as a characteristic feature of COVID-19. This conclusion was based on a previous study that assessed the use of CRegPs expression levels of PBMC cell populations, as markers of viral infection and reported no difference of CD55 expression levels in monocytes, neutrophils or lymphocytes [100] of patients with viral infection compared to healthy controls. Detsika et al. further demonstrated a negative correlation of increased CD55 levels in PBMCs of severely ill COVID-19 patients and the expression of type-I IFN response genes. This finding indicated a possible role of CD55 in the suppression of type-I IFN responses in COVID-19 patients and therefore in COVID-19 progression [99]. Yan et al. also based on single cell sequencing data analysis, identified distinct signatures of complement activation in myeloid, lymphoid, and epithelial cells from BALF samples of COVID-19 patients with severe disease [101]. This study demonstrated increased *C3aR1* and *CD46* expression in myeloid and lymphoid BALF cells respectively, from severely ill COVID-19 patients. Furthermore, the study identified increased expression of specific genes associated with the interferon-janus kinase (JAK)-1/2- signal transducer and activator of transcription (STAT)-1signaling system which were shown to be regulated by *C3aR1* and *CD46* [101]. A recent study further investigated the role of C1q and C4bp, in SARS-CoV-2 infection [102]. The study demonstrated the ability of C1q and C4b-binding protein to bind to SARS-CoV-2 pseudovirus, which resulted in viral entry inhibition as well as in amelioration of the SARS-CoV-2-mediated pro-inflammatory response by C1q and C4b-binding protein treatment of A549 cells expressing human angiotensin-converting enzyme-2 (ACE2) and transmembrane protease, serine 2 (TMPRSS2) in-vitro [102]. Taken together, the above-mentioned observations point to a pivotal role of complement activation in COVID-19 infection-mediated ARDS and the immune response driving disease progression, while further research in other viral mediated-pneumonias will enable the dissection of similar complement-driven mechanisms in an effort to further assess the role of the complement system in lung disease.

## Chronic lung pathologies

### COPD

COPD is a chronic condition encountered in the lungs characterized by airflow limitation, mainly resulting by inflammation of the airways [103]. Patients with COPD showed multiple episodes of symptoms exacerbation, usually triggered by respiratory infections, followed by periods of regression. Such exacerbations are characterized by an acute aggravation of chronic inflammation, and they are usually accompanied by activation of complement cascade.

As expected, given the fact that lungs in COPD are characterized by a continuous inflammatory state, a number of complement factors (C3a, C4a, C5a) have been found to be increased in both the sputum and plasma of patients with “stable” COPD [104]. In addition, several studies have revealed a further increase of sputum C3a and C5a during COPD exacerbations, compared to related baseline levels during periods of disease “stability”, while their respective levels seem to be directly associated with the severity and duration of the episodes [105, 106]. A reduction of CD46 levels was also reported to correlate with loss of lung function in COPD patients thus indicating a contributory role in the increased levels of complement observed in these patients [107]. On the other hand, a reduction in C1q levels has also been reported in serum samples of COPD patients compared to non-COPD controls [108]. C1q levels positively correlated with the forced expiratory volume in the first one second/forced vital capacity (FEV1/FVC) ratio and predicted FEV1%, thus adding to its potential as a disease progression marker. Finally, C3 increase was confirmed in a mouse model of cigarette smoke [109]. This study further showed that C3 knockdown exacerbated oxidative stress and apoptosis in 16HBE cells exposed to cigarette smoke, indicating thus a protective effect of C3 in COPD [109].

### Asthma

Asthma is considered to be a chronic inflammatory airway disease, mainly initiated by a hypersensitivity reaction of the airways that involves the immune system, against various environmental or other normally harmless, factors [110–112]. Given the important and multi-dimensional role of complement in both innate and adaptive immune networks, its functional significance in the context of allergic inflammation, including asthma, has been the subject of thorough experimental studies. Complement role in asthma is mainly mediated by anaphylatoxins C3a and C5a. Anaphylatoxin receptors are normally expressed by various cell populations of both human and murine lung tissue, including endothelial, epithelial and smooth muscle cells, while they are also found on cells of myeloid lineage, such as eosinophils, macrophages, dendritic cells and mast cells [113].

Increased C3aR and C5aR1/R2 expression has been confirmed during asthma in in-vivo asthma models, generated via treatment of mice with inhaled allergens, such as ovalbumin (OVA) and LPS. Challenge of mice with both factors lead to an upregulation of C3aR on both smooth muscle and bronchial epithelial cells, while C5aR expression was increased only on bronchial epithelium [114]. In the same murine asthma models an increase of both anaphylatoxin receptors was observed in resident as well as infiltrating leukocytes [115]. Further experimental studies have shed light into the function of complement in the generation of lung inflammation and airways hypersensitivity-mediated constriction. Challenge of mice lacking C3 (*C3*^−/−^) or C3aR (*C3aR*^−/−^) with OVA or other allergens have demonstrated the C3a/C3aR axis as an important contributor of the effector phase of asthma [116, 117]. In different murine models of asthma, deletion of C3 or C3aR resulted in a restrictive effect on airways hypersensitivity, albeit with contradictory data regarding their role in the inflammatory reaction, that leads to asthma development [116, 117]. One of the two studies involved use of *C3*^*−*/−^ mice and administration of OVA, or an *Aspergillus fumigatus* derived allergen, and reported a reduction in eosinophils and IL-4-producing cells, as well as attenuated Ag-specific IgE and IgG1 responses in *C3*^*−*/−^ mice compared with controls [116]. The other study involved *C3*^*−*/−^ mice with asthma triggered by exposure to airborne particulate matter and reported no difference in the inflammatory response between *C3*^*−*/−^ mice and controls [117]. C3aR genetic ablation was further shown to have a selective suppressive impact on airways’ constriction, without affecting inflammatory infiltrates which remained similar between *C3aR*^*−/−*^ animals and controls [118]. Finally, genetic ablation of anaphylatoxins’ receptors, C3aR and C5aR1/C5aR2 was found to result in a partial rescue of asthmatic phenotype in various *in-vivo* models of exogenous allergens administration, including reduction of inflammation and suppression of airways’ hypersensitivity constriction [118–120].

In addition, another investigation revealed that intratracheal administration of aerolized OVA in either C5aR null, or WT mice, under pharmacological blockade of C5aR, lead to a more robust sensitization phase compared to control animals, characterized by an enhanced Th2 response, significantly elevated serum IgE levels and substantially increased lung eosinophilic-neutrophilic infiltrates [121]. The above findings are indicative of an inflammatory-restrictive role of C5aR during the sensitization phase of asthma which is probably related to a suppressive function in activation of antigen-presenting cells. Indeed, the vigorous production of enhanced T helper 2 (Th2) cytokines after C5aR genetic or pharmacological alleviation, was associated with an enhanced function of a specific dendritic cell (DCs) subset, termed as myeloid DCs (mDCs) [122]. Furthermore, it has been shown that *C5aR1*^−^ DCs are characterized by increased levels of major histocompatibility complex-II (MHC-II) and co-stimulatory ligand CD40 compared to their C5aR positive counterparts, in a model in which allergen-induced autocrine C5a generation and subsequent C5aR1 activation in pulmonary CD11b^+^ cDCs promoted tolerance towards aeroallergens through downregulation of CD40 [123]. The role of C5a inhibition has been elucidated in animal model studies. C5a pharmacological blockade with PMX205 was shown to have a suppressive and anti-inflammatory effect in both the initial exposure to antigen (sensitization), as well as during the re-challenge (effector) phase, as defined by the restriction of neutrophilic and eosinophilic inflammatory infiltrates and the diminishment of Th2 type of response, with no effect in the airway responsiveness [124]. On the other hand, inhibition of C5 by a monoclonal antibody, during the effector phase in a house dust mite asthma model lead to a significant impairment of airways constriction, with no significant difference on lung histological alterations, including recruitment of inflammatory cells [124]. The levels of C3a, C4a and C5a and their respective receptors increase abruptly in asthma patients, as shown in sputum samples, BALF, or protein extracts of lung tissues from asthmatic patients compared to healthy controls [104, 125]. Moreover, increased levels of circulating C3a have been detected during asthma exacerbations, while polymorphisms of C3a and C3aR have been found to increase susceptibility to asthma development [126].

### ILDs

ILDs are a broad term used to describe a large spectrum of pulmonary diseases, which mainly affect the interstitial compartment of lung parenchyma, namely the tissue separating alveoli, leading to a compromise of gases exchange and consequently to a respiratory failure [127, 128]. Despite sharing the exclusive involvement of interstitial lung tissue, they are characterized by considerable heterogeneity regarding both their exact pathogenetic routes, morphological patterns and clinical course, with some displaying an acute and partially reversible damage, while others demonstrate a chronic and progressively aggravating injury [129]. Regarding their pathogenesis, ILDs could be categorized to two classes, according to the stimulus triggering their initiation. The first category, attributes ILDs to the susceptibility of lung tissue to the harmful effect of various environmental, toxic or infectious factors, while the second category treats ILDs as one of the multi-organ manifestations, encountered in systemic autoimmune conditions, such as systemic sclerosis, rheumatoid arthritis and sarcoidosis [130].

Evidence for complement involvement in disease pathogenesis emerged when polymorphisms of CR1 were detected in patients with sarcoidosis and idiopathic pulmonary fibrosis [131] while in a proteomic analysis of BALF from sarcoidosis patients, complement factors C3, C1 and factor B were found highly upregulated [132]. In addition to increased levels of complement fragments C3d, C4d and, factor B active fragment, Ba were also detected. Ba was further associated with different clinicopathological parameters of the disease, such as duration, dyspnea, fibrosis score, CRP and FVC, thus rendering it a potential marker of disease prognosis and progression [133]. Similarly, increased expression of various complement-related genes has been identified in a large cohort of patients with different forms of ILDs, such as idiopathic pulmonary fibrosis and sarcoidosis, in both BALF and plasma samples, including C5a, C4d, ficolin-2 and ficolin-3 [134]. In a similar context C1q was found highly upregulated compared to control lungs, in a gene expression profile analysis study of patients with idiopathic pulmonary fibrosis [135]. A similar large-scale gene expression study, conducted in patients with interstitial pneumonia identified complement related molecules, namely factors B, H and I as possible mediators of disease progression. Additional evidence supporting the causal relationship between ILDs and complement activation is provided by studies of protein semi-quantification, conducted via both western blot and immunohistochemical methods, which have confirmed the downregulation of complement inhibitory proteins, such as CD55, CD46, in alveolar and airway epithelium of such patients [136].

The potential of complement factors as disease progression markers was also shown in a study involving patients with the chronic vascular disease, pulmonary arterial hypertension (PAH) in which the altered levels of factors B and D allowed for their use, in combination with other proteins, in specific algorithms for prediction of disease progression [137] while upregulated expression of C3 protein was shown in a proteomics study on idiopathic PAH sera samples [138]. A recent study identified the alternative complement pathway as a key mediator of PAH using both samples from PAH patients as well a mouse model of pulmonary hypertension in mice deficient for various complement factors, C3, C5 and factor H [139].

### Lung cancer

The complement system has recently been recognised as an important regulator of cancer immunity [140]. The series of events needed to take place in order to have an effective immunological response against cancer, also referred to as the cancer immunity cycle [141], were described in detail and allowed to hypothesize when and how the complement system may act as a positive or negative regulator of the anti-cancer immune response. Since then, numerous studies have targeted the complement system in order to unravel its contribution to the anti-cancer immune response, and recently blocking of complement was added as a therapeutic scheme against lung cancer (STELLAR-001) [142]. In this context, Bulla et al. demonstrated that WT mice exhibited early deposition of C1q, higher vascular density and increased lung metastases compared with *C1qa*^−/−^ mice [143], thus identifying C1q as a tumour promoting factor.

The use of animal models has enabled further insight into the mechanisms by which complement is involved in lung tumour generation and progression. Initial evidence on the tumour promoting potential of the complement cascade was provided by Coralles et al. [144]. The study utilised a C5a antagonist and revealed slower progression of tumours in cancer bearing mice. Furthermore, it was shown that although C5a did not modify cancer cell proliferation *in- vitro*, it induced endothelial cell chemotaxis, blood-vessel formation and favoured the generation of the immunosuppressive microenvironment required for tumour growth by promoting myeloid-derived suppressor cells and immunomodulators, namely arginine-1 (ARG1), cytotoxic T-lymphocyte associated protein 4 (CTLA-4), IL-6, IL-10, lymphocyte-activation gene 3 (LAG3) protein and programmed death-ligand 1 (PDL1) [144].

In another elegant study by Kwack et al. the crucial role of C3 in lung cancer was confirmed. Using WT and *C3*^-/-^ mice the study revealed elevated levels of C3a in mice with lung cancer [145] and restriction or even complete absence of tumour growth. This growth inhibitory effect was reversed in mice which were depleted of CD4^+^ T cells, while administration of a C3a and a C5a antagonist also inhibited tumour growth. Consequently, this study provided direct evidence of the tumour promoting effect of the complement system [145].

Based on the above, a question of whether inhibition of complement could enhance anti-cancer therapies arises. This question was addressed successfully by Ajona et al. who demonstrated an additive anti-tumour effect by administration of a combination of anti-programmed cell death protein (PD) -1 and C5a blocking antibody in mice with lung cancer. The study revealed that blocking of PD-1 and C5a enhanced growth retardation and markedly extended survival in syngeneic lung cancer mouse models, a fact associated with CD8^+^ T cell activation [146]. A recent study identified C3 as a key mediator of paclitaxel resistance in mice with lung cancer receiving paclitaxel chemotherapy [147].

Another tumour promoting effect of the complement system has been described through the previously mentioned CRegPs, which inhibit the activation of the complement system. Increased expression of CD55, CD46 and CD59 has been documented in lung cancer cells [148] while another study showed increased factor H mRNA in lung cancer biopsies [149]. Increased secretion of factor I by non-small cell lung cancer (NSCLC) cells in-vitro has also been described. The overexpression of CRegPs by cancer cells is thought to allow evasion from the complement system and therefore cell lysis and destruction.

Increased complement activation has been described in cancer patients. Elevated C4d levels were detected in both BALF and plasma samples from lung cancer patients, and C4d plasma concentrations were proposed as a discriminatory characteristic between benign and malignant tumours [150]. An increase in C4 levels and its association with poor prognosis was reported in a recent study [151]conducted in patients with small cell lung cancer, which also identified the prognostic value of factor H, as its low levels were correlated with a high mortality risk [151]. In another study involving proteomic analysis of plasma samples from lung cancer patients, C3 and C4 protein levels were found elevated in comparison to healthy individuals [152], while Oner et al. also reported high levels of C3 and C4 in serum samples of lung cancer patients [153]. C5a anaphylatoxin levels were also reported to be significantly elevated in plasma samples from patients with NSCLC [144].

Increased complement activation has in addition been identified in the tumour microenvironment of patients with lung cancer. Gu et al. reported increased expression of C5aR in tumour cells isolated from tissue biopsy samples from patients suffering from NSCLC [154]. On the other hand, the low levels of C3 deposition in tissue from patients with NSCLC have been reported as an independent poor prognosis marker [155]. In a similar context, another study identified increased C1q deposition in lung tissue from lung adenocarcinoma, cancer patients [144]. The study described the increased deposition of C1q compared to other complement factors such as C4. The increased C1q deposition was also reported in a subsequent study on human malignant pleural mesothelioma biopsy samples which also suggested the tumour promoting role of C1q [156].

## Therapeutic potential of complement targeted therapies

Taking into consideration the involvement of complement in the pathogenesis of various diseases has prompted efforts towards targeting complement pathway related molecules for the development of novel therapies. The three different pathways and the plethora of molecules involved in this complex cascade further enable targeting of various components at both the final step, such as the inhibition by the well-known C5 inhibitor eculizumab, as well as targets upstream positions such as the C3 level. Recently, the interest of pharmaceutical companies in developing complement-related therapeutics has grown, and currently there are multiple different compounds and agents designed for the efficient blocking or competition of numerous complement components [157]. Complement related therapeutic strategies were employed recently during the COVID-19 pandemic for the treatment of this disease and are still under current investigation as combination therapies against various forms of lung cancer. The complement-related therapeutic agents which have been tested or are still under investigation against lung disease are summarised in Table 1.

**Table 1:** Complement-targeted therapeutics in lung disease

Name	Target	Type	Indication	Company	References
Eculizumab (Soliris®)	C5	mAb	COVID-19	Alexion	[157–159]
Vilobelimab	C5a	mAb	COVID-19, cutaneous squamous cell carcinoma	InflaRx	[156, 163, 164]
AMY-101	C3	compstatin peptide	COVID-19	Amyndas Pharmaceuticals	[160–162]
Narsoplimab	MASP-2	mAb	COVID-19	Omeros	[165]
Avdoralimab	C5aR1	mAb	Solid tumours, non-small cell lung cancer	Innate Pharma	[142]

mAb: monoclonal

Several complement targeted therapies were tested in the recent pandemic during which the need for fast and efficient regiments was imminent. Eculizumab, already approved for the treatment of paroxysmal nocturnal hemoglobinuria (PNH), was initially tried in critically ill COVID-19 patients and revealed an improvement of ARDS by reduction of oxygenation needs and inflammation, as well as a reduction of hospitalisation duration when used alone [158, 159] or in combination with the JAK1-2 inhibitor ruxolitinib [160]. Similarly, promising data were generated by administration of a C3 inhibitor, namely AMY-101, the administration of which in COVID-19 patients with severe ARDS safely reduced inflammation and ameliorated the syndrome [161, 162]; however, the proportion of patients with improved oxygenation did not reach statistical significance in larger trials [163]. The use of the C5a inhibitor vilobelimab in intubated COVID-19 patients improved survival [164] and was recently approved by the FDA for emergency use in COVID-19 hospitalised adults [165]. A lectin pathway inhibitor was also tested for its efficacy in treatment of COVID-19 patients with severe disease. Administration of narsoplimab, a MASP-2 inhibitor, was associated with a sustained decrease of inflammatory markers and a reduction of mortality [166] while results from larger ongoing trials are anticipated.

The important role of both C3 and C5a in lung cancer progression as shown by elegant studies using mouse models of NSCLC [144, 145] and the additive effect of complement inhibition in tumour progression [146] has provided strong evidence for complement related anti-tumour effect, thus possibly opening new therapeutic avenues. Furthermore, Yuan et al. used a C5aR1 inhibitor in mice with NSCLC under radiotherapy and showed an improved sensitivity and tumour response to radiotherapy, associated with increased expression of C5aR1 expression in CD8^+^ cells [167]. Taken together the above studies point towards novel therapeutic strategies involving complement inhibitors in combination with current schemes. This hypothesis has provided the ground for and is currently tested in a clinical trial in which the anti-C5aR1 antibody IPH5401, avdoralimab, is being evaluated in combination with the anti-PDL-1 antibody durvalumab in patients with solid tumours (STELLAR-001) [142]. Furthermore, the recently emerged C5a inhibitor, vilobelimab, is currently tested in a phase II clinical trial in combination with PD-1 checkpoint inhibitor, pembrolizumab, in cutaneous squamous cell carcinoma [157].

## Conclusions

Complement activation has been confirmed in the majority of lung diseases while the exact underlying mechanisms of complement involvement, in driving disease manifestation and progression, are not well understood. Given the diverse array of targets on the complement cascade, delineating these mechanisms is crucial in order to develop novel therapeutic strategies against lung diseases which remain a serious global health burden.

## Data Availability

All data are available within the manuscript.

## Abbreviations

ARDS: Acute respiratory distress syndrome
ACE2: Angiotensin-converting enzyme-2
ARG1: Arginine-1
BALF: Bronchoalveolar lavage fluid
COPD: Chronic obstructive pulmonary disease
CAP: Community acquired pneumonia
C1q: Complement component 1q
CR1: Complement receptor type 1
CRegPs: Complement regulatory proteins
COVID-19: Coronavirus disease
CRP: C-reactive protein
CTLA-4: Cytotoxic T-lymphocyte associated protein 4
DCs: Dendritic cell
FEV1: Forced expiratory volume in the first one second
FVC: Forced vital capacity
ICU: Intensive care unit
IFN: Interferon
IL: Interleukin
JAK: Janus kinase
*K. pneumoniae*: *Klebsiella pneumoniae*
LAG3: Lymphocyte-activation gene 3
MHC-II: Major histocompatibility complex-II
MBL: Mannan-binding lectin
MASPs: MBL-associated serine proteases
MAC: Membrane attack complex
MCP: Membrane co-factor protein
MERS-CoV: Middle east respiratory syndrome coronavirus
NETs: Neutrophil extracellular traps
NSCLC: Non-small cell lung cancer
OVA: Ovalbumin
PBMCs: Peripheral blood mononuclear cells
PNH: Paroxysmal nocturnal hemoglobinuria
PRPs: Pattern recognition proteins
PDL1: Programmed cell death-ligand 1
PD-1: Programmed cell death protein 1
PAH: Pulmonary arterial hypertension
RSV: Respiratory syncytial virus
SARS-CoV: Severe acute respiratory syndrome coronavirus
STAT-1: Signal transducer and activator of transcription
*S. pneumoniae*: *Streptococcus pneumoniae*
Th2: T helper 2
TMPRSS2: Transmembrane protease, serine 2
WT: Wild type
